# *Cis*-Segregation of c.1171C>T Stop Codon (p.R391*) in *SERPINC1* Gene and c.1691G>A Transition (p.R506Q) in *F5* Gene and Selected GWAS Multilocus Approach in Inherited Thrombophilia

**DOI:** 10.3390/genes12060934

**Published:** 2021-06-18

**Authors:** Donato Gemmati, Giovanna Longo, Eugenia Franchini, Juliana Araujo Silva, Ines Gallo, Barbara Lunghi, Stefano Moratelli, Iva Maestri, Maria Luisa Serino, Veronica Tisato

**Affiliations:** 1 Department of Translational Medicine, University of Ferrara, 44121 Ferrara, Italy; giovanna.longo@unife.it (G.L.); juliana.silva@unife.it (J.A.S.); ines.gallo@unife.it (I.G.); 2 Centre Haemostasis & Thrombosis, University of Ferrara, 44121 Ferrara, Italy; eugenia.franchini@unife.it (E.F.); stefanomoratelli@libero.it (S.M.); maria.luisa.serino@unife.it (M.L.S.); 3 University Center for Studies on Gender Medicine, University of Ferrara, 44121 Ferrara, Italy; veronica.tisato@unife.it; 4 Department of Life Sciences and Biotechnology, University of Ferrara, 44121 Ferrara, Italy; barbara.lunghi@unife.it; 5 Department of Medical Sciences, University of Ferrara, 44121 Ferrara, Italy; iva.maestri@unife.it; 6 Department of Translational Medicine and LTTA Centre, University of Ferrara, 44121 Ferrara, Italy

**Keywords:** *SERPINC1*, *F5*, FV Leiden, GWAS, *cis*-segregation, crossing-over, inherited thrombophilia

## Abstract

Inherited thrombophilia (e.g., venous thromboembolism, VTE) is due to rare loss-of-function mutations in anticoagulant factors genes (i.e., *SERPINC1*, *PROC*, *PROS1*), common gain-of-function mutations in procoagulant factors genes (i.e., *F5*, *F2*), and acquired risk conditions. Genome Wide Association Studies (GWAS) recently recognized several genes associated with VTE though gene defects may unpredictably remain asymptomatic, so calculating the individual genetic predisposition is a challenging task. We investigated a large family with severe, recurrent, early-onset VTE in which two sisters experienced VTE during pregnancies characterized by a perinatal in-utero thrombosis in the newborn and a life-saving pregnancy-interruption because of massive VTE, respectively. A nonsense mutation (CGA > TGA) generating a premature stop-codon (c.1171C>T; p.R391*) in the exon 6 of *SERPINC1* gene (1q25.1) causing Antithrombin (AT) deficiency and the common missense mutation (c.1691G>A; p.R506Q) in the exon 10 of *F5* gene (1q24.2) (i.e., FV Leiden; rs6025) were coinherited in all the symptomatic members investigated suspecting a *cis*-segregation further confirmed by STR-linkage-analyses [i.e., *SERPINC1* IVS5 (ATT)_5–18_, *F5* IVS2 (AT)_6–33_ and *F5* IVS11 (GT)_12–16_] and *SERPINC1* intragenic variants (i.e., rs5878 and rs677). A multilocus investigation of blood-coagulation balance genes detected the coexistence of FV Leiden (rs6025) in *trans* with FV HR2-haplotype (p.H1299R; rs1800595) in the aborted fetus, and *F11* rs2289252, *F12* rs1801020, *F13A1* rs5985, and *KNG1* rs710446 in the newborn and other members. Common selected gene variants may strongly synergize with less common mutations tuning potential life-threatening conditions when combined with rare severest mutations. Merging classic and newly GWAS-identified gene markers in at risk families is mandatory for VTE risk estimation in the clinical practice, avoiding partial risk score evaluation in unrecognized at risk patients.

## 1. Introduction

Inherited thrombophilia is considered a polygenic and multifactorial disease, where rare and common inherited prothrombotic defects combine with acquired/transient risk factors to finely tune disease penetrance, age of onset, and severity [1,2,3,4,5,6,7,8]. It is defined as a disorder of blood coagulation characterized by a tendency for thrombus formation in veins or arteries mainly due to anomalies in blood composition, blood flow, or vascular wall. Thrombophilia is often used to address venous thromboembolism (VTE) including deep vein thrombosis (DVT) and pulmonary embolism (PE). It is a common disease, characterized by an annual incidence of 1–3 patients per 1000 per year [9]. VTE has a mean case-fatality rate of 6.4% after a first VTE event being twice for PE (9.7%) than for DVT (4.6%) [9]. Both sexes are equally affected by a first VTE event, though males have more than 2-fold higher risk for recurrence and this has been considered in the past as a paradox also accounting for genetics and X- or Y-linked mutations within genes with sex-specific effects as for other complex diseases [10,11,12,13,14]. As research and knowledge in complex disease improve, novel risk factors can be identified, and new tests become available. Accordingly, to better predict the risk of VTE in carrier subjects, multilocus genetic risk score approaches based on Genome Wide Association Studies (GWAS) have been generated [15,16]. In the last few years, GWAS have made a massive effort in recognizing several common gene variants and low-penetrance gene variants (often Single Nucleotide Polymorphisms, SNPs), sometimes identifying unsuspected genes as contributors to the VTE global genetic risk score (GRS) [15,16,17]. Apart from coagulation and anticoagulation genes, platelets and other blood cells genes (e.g., those of the immune system cells) are also involved in inherited thrombophilia. GWAS, especially in exome (WES), and Transcriptome Wide Association Studies (TWAS) must be considered for a targeted and complete GRS assessment [18].

Among the acquired circumstantial conditions for VTE, pregnancy, hormone therapy, cancer, bone fractures, and immobilization are the strongest risk factors [19]. Since the heritability of VTE is over 60%, together with the classical rare gene defects of the natural anticoagulants (i.e., *SERPINC1*, *PROC*, *PROS1*), the conventional recognized risk models are based on the commonest prothrombotic mutations [20,21]. Accordingly, also considering the common *F5* (Factor V Leiden, c.1691G>A; rs6025) and *F2* (Prothrombin, c.20210G>A; rs1799963) gene defects, the approach only partially accounts for the high VTE incidence [21], suggesting that the risk assessment could be strongly improved including additional gene markers [20,22] to progress the guide for clinical geneticists in the management of inherited thrombophilia [21]. This approach should also be considered in a sex-oriented perspective due to the specific acquired and circumstantial risk situations the different sexes experience lifelong [23].

The *SERPINC1* gene (locus 1q25.1) spans over 13 kb and contains 7 exons encoding for the antithrombin (AT) serine protease inhibitor [24]. AT plays a crucial role as natural anticoagulant within the blood coagulation cascade, exerting its role mainly by inhibiting the serine protease thrombin (Factor IIa) and the activated factor X (FXa). *SERPINC1* gene defects are considered the strongest inherited risk factors for VTE so that AT homozygous deficiency causes embryonic death and the combination of different *SERPINC1* mutations or variants is responsible for the recently fascinating described genotype-phenotype gradient, that is, the clinical variability found among carriers of the same or different combination of *SERPINC1* defects [25,26,27]. The evidence that *SERPINC1* gene is poorly polymorphic underlies its high susceptibility even to minor changes in nucleotide sequence responsible for loss of function mutations and strong structural and functional changes, leading to severe thrombosis [28]. AT deficiency is indeed a rare thrombotic defect increasing the risk of thrombosis up to 10-folds in heterozygous carriers [24,29].

The *F5* gene (locus 1q24.2) encodes for coagulation FV, it spans about 75 kb and contains 25 exons [30]. FV has a two-faced role within the blood coagulation, acting either as procoagulant factor when it is activated (FVa) by thrombin (FIIa) or as an anticoagulant molecule after cleavage by activated protein C (APC) causing the loss of its procoagulant property. Accordingly, mutations in *F5* gene may result either in hemorrhagic or thrombotic clinical phenotypes [30]. The most common inherited prothrombotic defect is a mutation in *F5* gene (c.1691G>A; rs6025), responsible for the APC-resistance phenotype, better known as FV Leiden (p.R506Q) [31,32]. The presence of *F5* c.1691A allele increases the risk of thrombosis of about 6-folds in heterozygous carriers and up to 80-folds in homozygotes [30]. In addition, particular haplotypes in *F5* gene [33] can be present in *trans* with the mutated *F5* c.1691A, affecting in those heterozygous subjects the anticoagulant side of the FV protein guaranteed by the normal *F5* c.1691G allelic counterpart, this due to a reduction in the expression of the normal FV allele (e.g., HR2; c.4070A>G, p.H1299R; rs1800595) [34] or to a complete absence of the normal FV allele expression (e.g., c.5279A>G; p.Y1702C; rs118203907) [35]. These not-so-rare conditions exacerbate in turn the prothrombotic clinical phenotype accomplished by the mutated *F5* c.1691A allele as in the condition known as pseudo-homozygous APC-resistance [36,37,38]. Finally, the *F5* gene also contributes to arterial thrombosis as demonstrated in a study aimed at exploring the relationship between *F5* gene variants and the occurrence of coronary artery disease [39].

Rare loss of function mutations in *SERPINC1* gene and the common *F5* c.1691G>A mutation can be coinherited in the same individual leading to a strong increase in the associated VTE risk [1,2,3]. In addition, *SERPINC1* and *F5* genes map closely in the same region of chromosome 1 and this might allow the combined defect to co-segregate in *cis* exacerbating severity and penetrance of inherited thrombophilia [1,2,3].

Here, we report a multilocus genetic risk investigation in a large family carrying in *cis* a combined FV Leiden and *SERPINC1* gene defect with high penetrance, early onset, and severe phenotypes, including severe DVT in two sisters during pregnancy characterized by a perinatal in utero unusual thrombotic event and a massive DVT, respectively, in spite of prophylactic antithrombotic therapy.

## 2. Material and Methods

### 2.1. Family and Study Design

The family investigated in this report belongs to a previously described Italian family [2] whose members have now been enrolled in a local project entitled “Multilocus Genetic Scores predictive for Venous Thromboembolism Risk (MaGiSTER): real life evaluation and validation in a cohort of VTE patients” aimed at scoring the VTE risk modification within the thrombophilic family characterized by well-established gene defects. The previous report did not recognize in the family the main gene defect(s) and did not perform a sequence analysis of the *SERPINC1* gene but just reported a phenotypic assessment of AT antigen and activity levels, and did not implement an investigation through a multilocus genetic approach nor evaluate any worsening of the clinical phenotypes. As far as the MaGiSTER study is concerned, it was approved by the local IRB (code number 242/2020) according to the Declaration of Helsinki and all the members of the investigated family signed informed consent at the time of the blood drawn.

### 2.2. DNA Extraction, PCR Conditions, and Sequencing

Whole blood was collected from the family members and frozen at −80 °C. DNA was isolated from thawed whole blood by the automated DNA extraction and purification robot (BioRobot EZ1 System QIAGEN; Hilden, Germany).

*SERPINC1* full-length gene amplicons were obtained by PCR of specific fragments including promoter, exons and exon-intron junctions by PCR-mediated direct sequencing by using the BigDye Terminator v1.1 Cycle Sequencing Kit and ABI Prism 310 Genetic Analyzer (Applied Biosystems, Waltham, MA, USA) according to previous reports [40].

The multilocus-genetic approach, including *ABO* rs8176719; *F2* rs1799963; *F5* rs6025; *F5* rs4524; *F5* rs1800595; *F11* rs2289252; *F11* rs2036914; *F12* rs1801020; *F13A1* rs5985; *SERPINE10* rs2232698; *SERPINC1* rs121909548; *FGG* rs2066865; and *KNG1* rs710446, was selected from previous informative multilocus studies on the prediction of the thrombotic risk [20,22,41]. Genotyping for each SNP was performed by *rhAmp*^®^ *SNP Assay* (IDT, Integrated DNA Technologies, Coralville, IA, USA) on QuantStudio^TM^ 3-Real-Time PCR System (Thermo Fisher Scientific, Waltham, MA, USA) according to the supplier’s instructions. Within each run, DNA samples with a known genotype were used as internal control references.

### 2.3. STRs Linkage Analyses

The sequence primers for PCR amplification of *SERPINC1* IVS5 STR (ATT)_5–18_ were as follows: P1(Fw): 5′-TGA AGC CTG AGA ATG AAT TAT CAG-3′; P2(Rev): 5′-AGA GTG GGG AAG GTG TAC TC-3′; and P3(Rev): 5′-CCA CTG CAC TCC AGC CTG GG-3′. The P3 primer was 6-FAM labelled at 5′ end. PCR amplification was performed in a total volume of 50 μL by using P1 and P2 (first PCR), and P1 and P3 (nested PCR), according to previous reports [42].

The sequence primers for PCR amplification of *F5* IVS2 STR (AT)_6–33_ were as follows: P1(Fw): 5′- GAT TGC TTG AGG CCA GGA GTT-3′; P2(Rev): 5′-TTG TCC TAA ATG ACC CTC TTG C-3′. The P1 primer was 6-FAM labelled at 5′ end. PCR amplification was performed in a total volume of 50 μL according to previous reports [43].

The sequence primers for PCR amplification of *F5* IVS11 STR (GT)_12–16_ were as follows: P1(Fw): 5′-GTG GGT GAC ATC ATA GC-3′; P2(Rev): 5′-TGA CAT GGA CTA TAA CAC-3′. The P1 primer was 6-FAM labelled at 5′ end. PCR amplification was performed in a total volume of 50 μL according to previous reports [44].

Primers and FAM labeling were from IDT (Integrated DNA Technologies, Coralville, IA, USA), TopTaq DNA polymerase was from Qiagen LLC (Germantown, MD, USA) and all the PCR amplifications were performed on an Agilent SureCycler 8800 system (Santa Clara, CA, USA) according to the supplier’s instructions.

By Denaturing Capillary Electrophoresis Analysis, one-tenth of each FAM-labelled PCR amplicon was diluted in HD-Formamide solution and GeneScan 500ROX Size Standard (Applied Biosystems, Thermo Fisher Scientific, Waltham, MA, USA). The samples were run on ABI Prism 310 Genetic Analyzer Instrument and analysed by GeneMapper^®^ Software 5.0 (Applied Biosystems, Thermo Fisher Scientific, Waltham, MA, USA). Within each run, DNA samples with known genotype were used as internal control references.

### 2.4. Restriction Analyses

By using the same couples of primers as in the sequence analyses, two common SNPs were further investigated to complete family linkage studies. The c.1011A>G (p.Q337Q) synonymous variant (rs5878) within the *SERPINC1* exon 5 detectable by PstI restriction analysis (NEB, Ipswich, MA, USA) common among Caucasians (ALFA Allele Frequency: A = 0.64; G = 0.36) and the g.14956C>G variant (rs677) within *SERPINC1* intron 6–7 detectable by DdeI restriction analysis (NEB, Ipswich, MA, USA) (ALFA Allele Frequency: C = 0.87; G = 0.13). Restriction analyses were performed according to the supplier’s instructions.

## 3. Results

### 3.1. Family History and Index Cases

**Figure 1:**
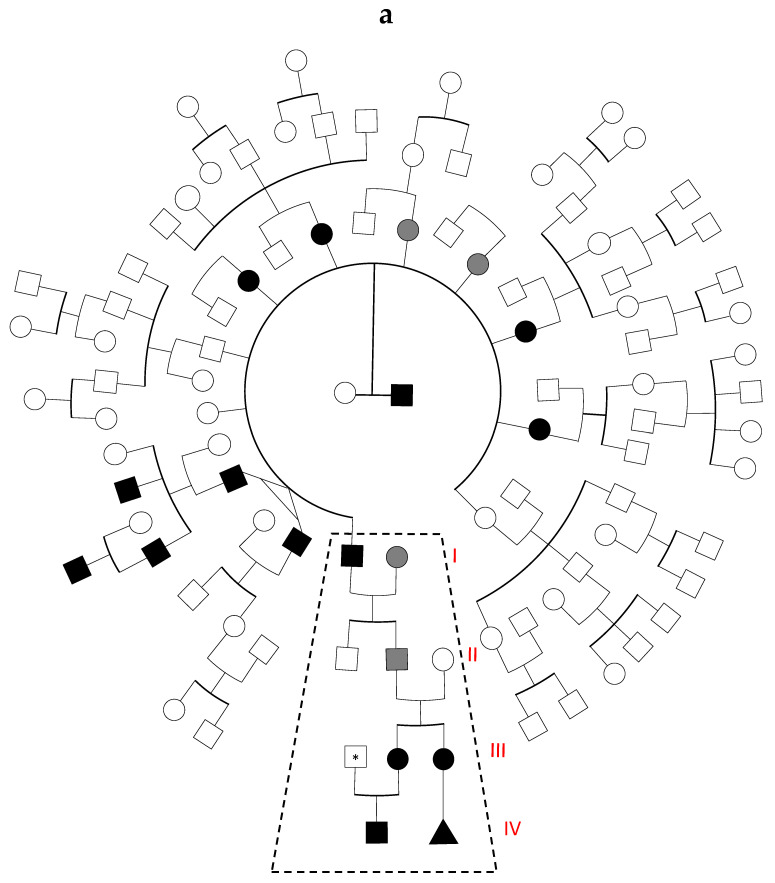

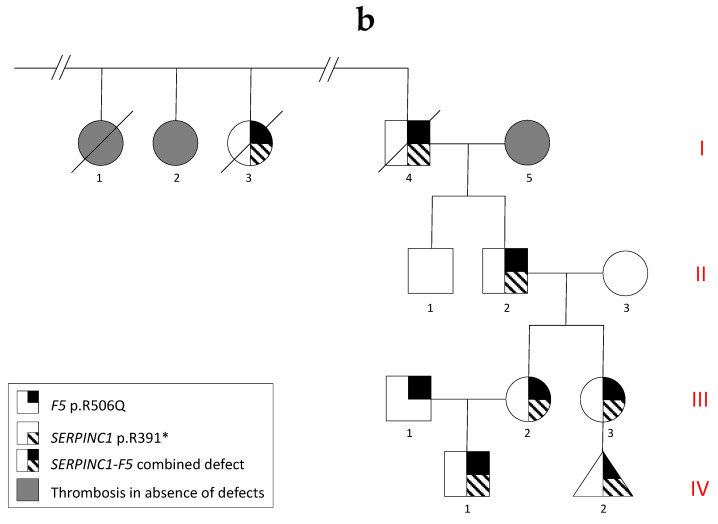
(**a**) Full pedigree of the original family. Black symbols refer to thrombotic subjects carrying the combined AT-FV Leiden defect (i.e., *SERPINC1*-*F5* mutations). Grey symbols refer to thrombotic subjects in the absence of any identified defect. The asterisk indicates an asymptomatic carrier of a single *F5* p.R506Q defect. The triangle symbol indicates aborted fetus. Dashed frame indicates subfamily described in Figure 1b. (**b**). Extended pedigree of the previous investigated family [2]. This is part of the full pedigree shown in Figure 1a (dashed frame). The different symbols are specified in figure legend (bottom left of the figure). The triangle symbol indicates aborted fetus. Strike-through symbols indicate dead individuals.

Figure 1a shows the original large family tree characterized by a relapsing of severe thrombotic and thrombophlebitis episodes at young age. Ten members belonging to the first generation had thrombotic manifestations with seven of them characterized by type-1 AT deficiency (AT level 45–50%; normal range, 70–125%). Thrombotic manifestations among the subsequent generations were clinically reported only within two branches of the family, and one of these was previously published as characterized by the coexistence of the type-1 AT deficiency and FV Leiden combined defect (dashed frame in Figure 1a) [2].

Figure 1b shows an extended family tree of the previously described family, in that report the propositus (I3) and her brother (I4) had their first DVT episode at the age of 20 and 21 years, respectively, characterized by following mixed arterial and venous (I3) and venous (I4) relapses in the presence of the combined AT-FV Leiden defect. Interestingly, although no prothrombotic defects were detected, additional members of the family had thrombotic manifestations: spontaneous DVT at age of 47 with relapse (I1), DVT after surgery at age of 45 (I2), and recurrent thrombophlebitis (I5). With regard to subject II2, at the age of 14 years, he had his first DVT after surgery and despite anticoagulant therapy he relapsed at the age of 20, experiencing bilateral DVT and successive saphenectomy. Moreover, his two daughters (III2 and III3), asymptomatic at the time of the first investigation [2], subsequently developed severe DVT episodes. At the age of 28 years, the first daughter (III2) during her first pregnancy (23 weeks of gestation) had DVT and afterward underwent full heparin treatment. Her son (IV1) developed severe perinatal vein renal thrombosis during the first week after birth and a further deep instrumental investigation confirmed the in utero origin of the renal thrombosis. At the age of 8 years, the second daughter (III3), in the absence of concomitant risk conditions had her first DVT and at the age of 15 years she developed abdominal aorta thrombosis. After two previous spontaneous miscarriages, at the age of 23 years, she had a massive VTE and a transient ischemic accident (TIA) during the last pregnancy. This complex thrombotic condition required premature life-saving pregnancy-interruption at 8 weeks of gestation, despite heparin treatment and negative results for autoantibody investigations [45]. To summarize, the AT-FV Leiden combined defect was detected in the following thrombotic subjects (i.e., I3, I4, II2, III2, III3, IV1). A laboratory screening in subject III1detected FV Leiden in heterozygous condition and this could better explain the severe in utero/perinatal thrombosis in the son (IV1), potentially at risk to be FV Leiden homozygotes in the presence of AT deficiency. Child IV1 was under heparin treatment until 18 months; he is now 4 years-old, and he did not inherit both FV Leiden alleles from his parents showing an AT-FV Leiden combined defect in heterozygous condition. He is now on lifelong oral anticoagulant therapy while his father is up to now completely asymptomatic.

The combined AT-FV Leiden defect was also indirectly detected in the aborted tissue (IV2) only by molecular analysis of DNA extracted from paraffin-embedded material (see below).

### 3.2. Genetic Analyses

In the affected members of the family, we detected a combined defect due to a type-1 AT deficiency caused by a previously described c.1171C>T mutation (CGA > TGA) in the exon 6 of *SERPINC1* gene causing a premature stop codon at arginine 391 (p.R391*) [28] and the common FV Leiden mutation due to an arginine-to-glutamine change at codon 506 (p.R506Q) in the exon 10 of *F5* gene.

Table 1 shows the list of the SNPs investigated and the genotyping results in the members of the family presented in Figure 1b. The carrier condition of the minor allele (i.e., hetero/homozygosis) for each SNP is shown in bold regardless it is considered the risk-allele and the reported ALFA frequency (Allele Frequency Aggregator; https://www.ncbi.nlm.nih.gov/snp/docs/gsr/alfa/, accessed on 12 June 2021) is shown in brackets (see MAF).

As regards the two sisters (III2 and III3), who experienced severely complicated pregnancy outcomes, apart from the combined *SERPINC1*-*F5* defect, they both were homozygous for *F11* rs2289252, heterozygous for *F12* rs1801020 and *F13A1* rs5985, and carried *KNG1* rs710446 in homozygous and heterozygous conditions, respectively.

As regards child IV1, who experienced the perinatal renal thrombosis and the aborted fetus IV2, apart from the combined *SERPINC1*-*F5* defect, they both carried *F12* rs1801020, *F13A1* rs5985 and *KNG1* rs710446 in heterozygous and *F11* rs2289252 in homozygous condition. In addition, the aborted fetus was diagnosed as a female by means of sex-specific PCR fragments (X/−, −/Y, and X/Y), and she carried *F5* HR2 rs1800595 in *trans* with *F5* Leiden rs6025 almost certainly inherited from her father (not shown in the figure and not available for the analysis). Unexpectedly, subject III1 carried isolated heterozygous *F5* p.R506Q mutation with a 50% chance of transmission to his son at risk to inherit one *F5* p.506Q-allele from both parents, then becoming a severely affected QQ-homozygotes in combination with the *SERPINC1* mutation. As explained above, this dramatic condition did not occur.

### 3.3. Family Linkage Analysis

**Figure 2:**
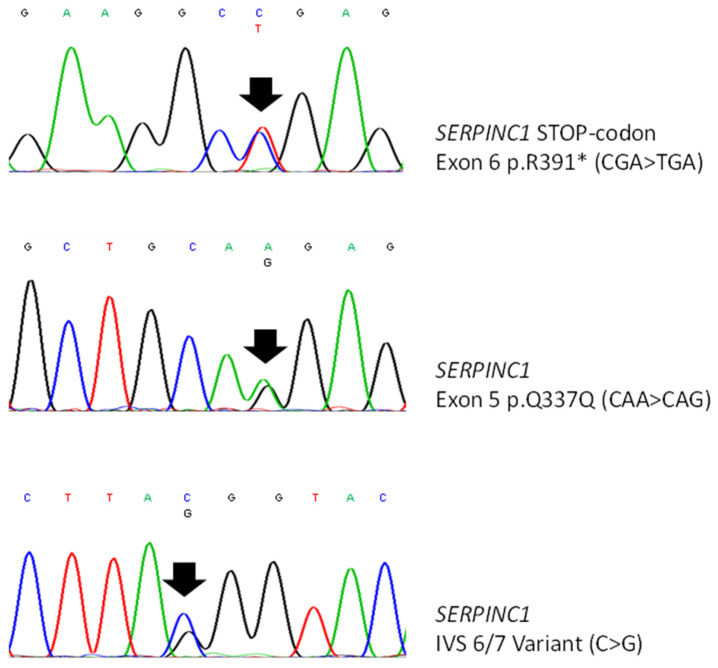
Sequence analysis of *SERPINC1* gene showing: p.R391* STOP-codon (upper panel); p.Q337Q synonymous variant (middle panel); intronic (IVS 6–7) C > G variant (rs677) (lower panel).

In all the members of the investigated family carrying the type-1 AT deficiency, the *SERPINC1* c.1171C>T stop codon (p.R391*) segregated with the c.1011A-allele of the synonymous p.Q337Q variant (rs5878), the g.14956C-allele variant (rs677), and the STR IVS5 (ATT)_12_ repeat in the *SERPINC1* gene (Figure 2, Table 2). The highly polymorphic trinucleotide STR yielded different heterozygous status in every affected member, allowing us to undoubtedly recognize the mutated *SERPINC1* allele along with the four generations.

Among FV Leiden carriers, subject II2 and his two daughters (III2 and III3) carried the *F5* IVS2 (AT)_17_ allele, and undoubtedly inherited from their mother (II3) the normal (AT)_19_ allele. On the contrary, child IV1 appeared to have inherited the FV Leiden allele from his carrier father (III1) suspecting an intragenic crossing-over event that cannot be confirmed by our investigation.

Finally, the known strong linkage between FV Leiden mutation and the *F5* IVS11 (GT)_14_ repeat allowed us to easily discriminate the normal *F5* counterpart allele in each subject along with the four generations (Table 2).

## 4. Discussion

Inherited thrombophilia is a multifactorial disorder in which genetic predispositions together with circumstantial risk factors interact to develop the clinical phenotype; therefore, acquired and genetic risk factors may often coexist in the same patient. Defects in the *SERPINC1* gene, responsible for AT deficiency, are considered the strongest inherited VTE risk factors, and the associated VTE incidence rate is about 1% per year among subjects carrying the single gene defect [46]. On the other hand, the coexistence of additional inherited or acquired risk predispositions synergistically increases the risk up to 10–20 fold, which is greater than the sum of their single risks.

It has been described that the mean onset age of the first VTE event among patients carrying a single *SERPINC1* gene defect is higher than that of patients carrying two or more thrombotic defects, and this is particularly evident among patients belonging to the same family [1,2,46]. Of particular interest is the coexistence of *SERPINC1* and FV Leiden defects in the same patient, characterized by two distinct familial inheritance patterns. In detail, both genes closely map on chromosome 1 (i.e., *F5*, 1q24.2, g.169.511.954–169.586.531 and *SERPINC1*, 1q25, g.173.903.804–173.917.378), and their gene defects can be either in the same (*cis*) or in separate (*trans*) copy of chromosome 1. In this study, the cosegregation in *cis* of the two defects (i.e., *F5*, c.1691G>A; *SERPINC1*, c.1171C>T) leads to the highest penetrance of familial thrombophilia and could be rarely terminated by recombinant events because of their relative short distance (g.169.549.811 and g.173.907.497 respectively). Moreover, it has been recently described an intriguing genotype-phenotype gradient of the clinical phenotype, accounting for the wide variability found among carriers of *SERPINC1* combined defects [27]. This concept could indeed also be extended to the coexistence of additional defects in different genes [20,22,41], which is the rationale of the polygenic nature of inherited thrombophilia and is part of the proposed MaGiSTER Study. Accordingly, the accumulation of mild unknown prothrombotic factors identifiable by GWAS might also be present in cases with a more severe clinical phenotype and contribute to the final individual risk.

During the drawing of the present manuscript, exhaustive counselling with the members of the presented family ascribed the severest clinical phenotype to subfamily 1b, mainly characterized by DVT during pregnancy in the two sisters despite a prophylactic full heparin treatment in which the younger also had several previous spontaneous miscarriages. Consistent with this synopsis, there is also the perinatal renal thrombosis in the fetus of the older sister.

A multilocus genetic approach, by selecting those SNPs previously shown to be significantly associated with thrombosis and/or with pregnancy-related thrombosis in GWAS or large multicenter studies, could reveal if additional molecular markers had a role in this peculiar clinical finding [47,48,49,50,51].

Interestingly, *F12* rs1801020, described as associated with circulating coagulation FXII levels and with arterial or venous thrombosis [52], was found in a heterozygous condition both in the two sisters and in their offspring (newborn and aborted fetus). A similar condition was detected for further at-risk genotypes marked as *F11* rs2289252 and rs2036914, associated with circulating coagulation FXI levels and with antenatal and pregnancy-related thrombosis [47,53,54] as well as *KNG1* rs710446, associated with in vitro coagulation test (aPTT) variability and with venous thrombosis [54].

Altogether, the above genes coding for proteins belonging to the High Molecular Weight Kininogen/Prekallikrein/FXI/FXII contact system, have a key role in blood coagulation balance and their defects are not associated with bleeding but rather with venous or arterial thrombosis and recurrent pregnancy loss also affecting fetal-placental unit as demonstrated by previous GWAS and case-control studies [52,53,54]. Although this is in line with their physiological role in regulating important vascular activities as vasodilatation and modulation of thrombus formation by anti-coagulant, anti-aggregant, and pro-fibrinolytic properties [55,56], large meta-analyses and GWAS revealed weak or null associations with thrombosis or cardiovascular diseases [57,58].

Particular consideration should be taken into account for *F5* gene, not only for its closeness with *SERPINC1* gene, allowing in the present family the coinheritance in *cis* of both defects, but also for the presence of additional at-risk SNPs within the *F5* gene that may modify the overall individual risk. As regards *F5* rs4524, the minor allele (T > C) has been described as protective from VTE in non-carriers of Factor V Leiden (rs6025) [59]. In addition, rs4524 has been consistently associated with VTE in three large control-case studies (i.e., LETS, “Leiden Thrombophilia” study; MEGA-1 and MEGA-2, “Multiple Environmental and Genetic Assessment of Risk Factors for Venous Thrombosis” study) also after adjustment for FV Leiden, being the two variants in low linkage disequilibrium (*r*^2^ = 0.02) [48,49]. As regards *F5* rs1800595, also known as FV HR2, the minor allele (A > G) is associated to decreased coagulation FV levels and the coinheritance with FV Leiden increases the VTE risk of about 10–15 fold and decrease the age of the first thrombotic events of about six years [34,35,36]. Moreover, infant renal thrombosis associated with *F5* rs6025 was previously reported, though a not satisfactory genetic investigation was performed [60]. We finally detected in the two sisters and in the new-born the at risk rs4524 genotype while in the aborted tissue the rs4524 and FV HR2 heterozygous conditions could have seriously worsened the already compromised coagulative balance as previously described [37,61].

## 5. Conclusions

The present study highlights that the coexistence of selected at-risk gene variants might contribute to the exact assessment of the individual thrombotic risk. The risk that severest genetic combinations might occur during generations needs to be properly considered and assessed also in families with well-established and identified genetic defects. In the OMIC era, the novel recognized genetic and acquired risk factors for thrombosis and pregnancy related complications [62,63,64,65,66] strongly prompt towards translational real-life applications, proposing that merging classic and newly GWAS- and/or TWAS-identified markers and circulating biomarkers in selected at risk populations is mandatory for a complete, personalized and sex-related risk assessment [12] to avoid partial risk score estimations in unrecognized at-risk patients.

## Figures and Tables

**Table 1:** Genotyping on selected SNPs in the members of Family 1b.

Ch.	Gene	Change (nt)	MAF	Variant	Change (aa)	Family 1b						
						II2	II3	III1	III2	III3	IV1	IV2
9	*ABO* rs8176719	−/G	G (0.379)	c.261delG	−	−/−	−/−	−/−	−/−	−/−	−/−	−/−
11	*F2* rs1799963	G > A	A (0.0135)	3′ UTR	−	GG	GG	GG	GG	GG	GG	GG
1	*F5* rs6025	G > A	A (0.025)	Missense	R506Q	G**A**	GG	G**A**	G**A**	G**A**	G**A**	G**A**
1	*F5* rs1800595	A > G	G (0.0485)	Missense	H1299R	AA	AA	AA	AA	AA	AA	A**G**
1	*F5* rs4524	T > C	C (0.267)	Missense	K830R	T**C**	TT	TT	TT	TT	TT	T**C**
4	*F11* rs2289252	G > A	A (0.399)	Intron	−	**AA**	**AA**	**A**G	**AA**	**AA**	**AA**	**AA**
4	*F11* rs2036914	G > A	A (0.479)	Intron	−	GG	GG	G**A**	GG	GG	GG	GG
5	*F12* rs1801020	G > A	A (0.237)	5′ UTR	−	GG	G**A**	G**A**	G**A**	G**A**	G**A**	G**A**
6	*F13A1* rs5985	G > T	T (0.243)	Missense	V34L	GG	G**T**	GG	G**T**	G**T**	G**T**	G**T**
1	*SERPINE10* rs2232698	G > A	A (0.0077)	Stop Gained	R67 *	GG	GG	GG	GG	GG	GG	GG
1	*SERPINC1* rs121909548	G > T	T (0.0015)	Missense	A384S	GG	GG	GG	GG	GG	GG	GG
4	*FGG* rs2066865	C > T	T (0.23)	near 3′UTR	−	CC	CC	CC	CC	CC	CC	CC
3	*KNG1* rs710446	T > C	C (0.419)	Missense	I581T	T**C**	T**C**	TT	**CC**	T**C**	T**C**	T**C**

Ch.: Chromosome; MAF: Minor Allele Frequency; G/G or −/G: likely to have blood type A or B; −/−: likely to have blood type O; the asterisk (*): indicates a translation termination codon (gained stop codon). The minor allele is shown in bold.

**Table 2:** Haplotyping in the members of Family 1b.

Gene	Variation	Family 1b						
		II2	II3	III1	III2	III3	IV1	IV2
*SERPINC1*	c.1171C>T (p.R391*)	C**T**	CC	CC	C**T**	C**T**	C**T**	C**T**
*SERPINC1*	c.1011A>G (p.Q337Q) rs5878	**A**A	AG	AG	**A**G	**A**G	**A**G	**A**G
*SERPINC1*	(rs677) C > G	**C**G	CG	GG	**C**G	**C**G	**C**G	**C**G
*SERPINC1*	IVS5 (ATT)_5–18_	10/**12**	11/13	10/11	11/**12**	11/**12**	10/**12**	nd
*F5*	c.1691A>G (p.R506Q)	A**G**	AA	A**G**	A**G**	A**G**	A**G**	A**G**
*F5*	IVS2 (AT)_6–33_	15/**17**	15/19	16/20	**17**/19	**17**/19	16/19	nd
*F5*	IVS11 (GT)_12–16_	**14**/15	13/15	13/**14**	13/**14**	13/**14**	13/**14**	14/**14**

nd: not determined due to shortage of genetic material from aborted sample (IV2). Mutated alleles, or those in linkage with the defect(s), are shown in bold.

## Data Availability

All relevant data are included in the manuscript.
